# Diversity, function, and transcriptional regulation of gut innate lymphocytes

**DOI:** 10.3389/fimmu.2013.00022

**Published:** 2013-03-04

**Authors:** Lucille Rankin, Joanna Groom, Lisa A. Mielke, Cyril Seillet, Gabrielle T. Belz

**Affiliations:** ^1^Division of Molecular Immunology, The Walter and Eliza Hall Institute of Medical ResearchMelbourne, VIC, Australia; ^2^Department of Medical Biology, University of MelbourneMelbourne, VIC, Australia

**Keywords:** innate lymphocytes, lymphoid tissue inducer cells, progenitor cells, transcription factors, differentiation, lymphoid tissue, immune protection

## Abstract

The innate immune system plays a critical early role in host defense against viruses, bacteria, and tumor cells. Until recently, natural killer (NK) cells and lymphoid tissue inducer (LTi) cells were the primary members of the innate lymphocyte family: NK cells form the front-line interface between the external environment and the adaptive immune system, while LTi cells are essential for secondary lymphoid tissue formation. More recently, it has become apparent that the composition of this family is much more diverse than previously appreciated and newly recognized populations play distinct and essential functions in tissue protection. Despite the importance of these cells, the developmental relationships between different innate lymphocyte populations remain unclear. Here we review recent advances in our understanding of the development of different innate immune cell subsets, the transcriptional programs that might be involved in driving fate decisions during development, and their relationship to NK cells.

## INTRODUCTION

Mucosal surfaces of the body are constantly bombarded with a variety of both innocuous and pathogenic organisms. Lymphocytes of the gut-, bronchus-, and nasal-associated lymphoid tissues (GALT, BALT, and NALT, respectively) play a critical role in protecting the body from harmful pathogens that enter through the mucosal tissues and lung.

**FIGURE 1:**
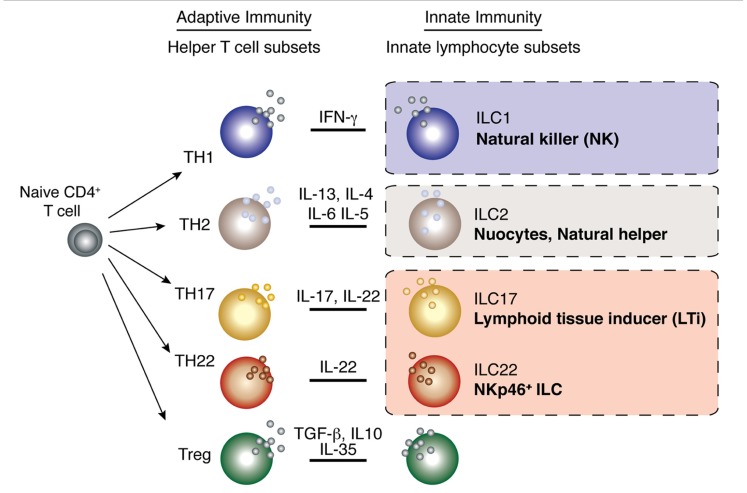
**Parallels between innate lymphocyte populations and CD4^+^ helper T cell subsets**. Multiple populations of CD4^+^ helper T cells exist and these are characterized by the production of signature cytokines by each subset. As the different populations of innate lymphocytes have emerged, it has become increasingly clear that these subsets produce an array of cytokines that parallel those of the helper T cell subsets.

Innate lymphoid cells (ILCs) are an expanding family of lymphocytes with innate cell characteristics. They co-ordinate the organization of lymphoid tissues, maintain epithelial tissue integrity, are responsible for the anatomical containment of commensal bacteria and play important roles in the protection against pathogens early during infection. Individual populations of ILCs display distinct cytokine signatures in a manner analogous to the specialization of cytokine secretion found in T helper (Th) cell subsets (**Figure 1**) which has come to define ILC subsets as innate versions, or perhaps as ancestors, of adaptive Th cells. Importantly, innate lymphocytes produce their effector cytokines in response to non-specific danger signals much more rapidly than their adaptive T cell counterparts allowing them to act immediately, before antigen specificity is acquired. This rapid induction of effector function allows ILCs to provide front-line protection at the onset of an immune response thereby limiting pathogen spread and regulating homeostatic tissue integrity.

**FIGURE 2:**
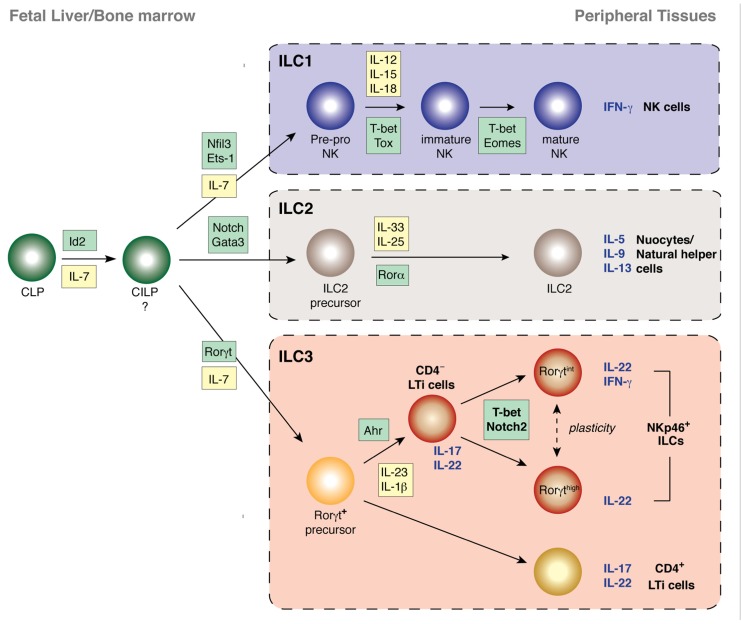
**Transcriptional programs controlling the development of innate lymphocyte populations from their progenitor cells in the fetus and adult**. The transcription factor Id2 is critical for the development of all NK cells and other ILC populations and is likely to direct the emergence of the common innate lymphoid progenitor (CILP) downstream of the common lymphoid progenitor (CLP) that does not express Id2. The first expression of Id2 from cells derived from the CLP marks the emergence of the pre-pro NK cells, the earliest progenitor of the NK cell lineage. LTi cells and NKp46^+^ ILCs all express and are dependent on Rorγt while ILC2 and NK cells develop without passing through a Rorγt-dependent stage. ILC2, however, rely on Rorα and Gata3 for their development, expansion, and survival following stimulation. CD4^+^ and CD4^-^ Id2^+^Rorγt^+^ LTi cells undergo diversification to generate terminally differentiated NKp46^+^ ILCs. CD4^+^ LTi cells appear to have already established a stable phenotype. In contrast, CD4^-^ LTi cells expand in response to various cues such as stimulation through AhR. Further environmental cues induce expression of T-bet and Notch ligands induce Notch signaling which results in the development of NKp46^+^ ILCs that produce abundant IL-22 and protect against bacterial infection.

The heterogeneity of ILCs has become increasingly complex. Initially, these cells were categorized based on their dominant expression of a single cytokine and confusingly, many different names have been applied to similar populations by the various research groups. As the diversity within the subsets has emerged, the need for consistent nomenclature has become apparent (Spits et al., 2013). It is now suggested that ILCs be grouped into three broad populations based on their phenotype, function, and transcriptional regulation (Tait Wojno and Artis, 2012; Spits et al., 2013; **Figure 2**). Group 1 ILCs (ILC1) are composed of the prototypical ILC, natural killer (NK) cells. This classification, however, might also include other ILCs that express the transcription factor T-bet and produce interferon-γ (IFN-γ; Spits and Di Santo, 2011; Spits and Cupedo, 2012). Group 2 ILCs (ILC2) produce the Th2 type cytokines interleukin (IL)-5 and IL-13 and are important for helminth expulsion. The group 3 ILCs (ILC3) produce Th17 type cytokines IL-17 and IL-22. They comprise the classical lymphoid tissue inducer (LTi) cells that are responsible for the generation of lymphoid tissue during embryogenesis, LTi-like cells that are phenotypically similar to LTi cells but are enriched in the intestine of the adult, together with the natural cytotoxicity receptor (NCR)-expressing NKp46^+^ ILC that produces IL-22 and not IL-17.

Within these groupings, ILC subsets share some overlapping features in their surface receptor phenotype and cytokine production but are distinct in their requirements for specific different transcription factors. Specification of immune cell fate is a stepwise process encoded by transcription factors that act on committed multi-potent progenitors and sequentially restrict their developmental fate to a particular lineage allowing them to develop into the mature cell type. Much has now been done in defining the regulatory circuits of B cells (Nutt et al., 2011), T cells (Kaech and Cui, 2012), NK cells (Sun and Lanier, 2011; Bezman et al., 2012), and dendritic cells (DCs; Belz and Nutt, 2012), but the lineage defining transcription factors required for the differentiation of innate lymphocyte subsets is less well understood. Understanding the transcription factors and transcriptional networks that control the differentiation of innate immune cell populations is a rapidly developing area of research.

All ILC subsets are dependent on the transcriptional regulator inhibitor of DNA binding 2 (Id2) while ILC3 cells or Rorγt^+^ ILCs rely on retinoic acid-related orphan receptor (Ror)γt downstream of Id2. A number of other transcription factors such as Rorα, nuclear factor IL-3 [Nfil3, also known as E4-binding protein 4 (E4BP4)], thymocyte selection-associated high-mobility group box protein (Tox), aryl hydrocarbon receptor (Ahr), Runt-related transcription factor (Runx)/CCAAT-binding factor (Cbfβ), Gata-binding protein 3 (Gata3), and Notch have also been implicated in the development, survival, and function of these populations.

As yet, the relationships between highly similar ILC populations such as the different subsets of Rorγt^+^ ILCs and whether developmental plasticity enables dynamic inter-conversion between ILC subsets in response to environmental stimuli is unclear. Nevertheless, the recent rapid progress of this research area promises a rich understanding of the complexity of the innate defense system protecting the body’s surfaces. Here we review the current knowledge of the transcription factors that regulate the features of ILCs (**Figure 2**; **Table 1**).

**Table 1:** Requirement of different transcription factors for lymphoid tissue and ILC development.

Transcription factor	NK cells (ILC1)	Nuocytes/natural helper cells (ILC2)	LTi cells (NCR^-^ ILC3)	NKp46^+^ ILCs (NCR^+^ ILC3)	Lymphoid tissue organogenesis
Id2	-	-	-	-	None (Yokota et al., 1999)
RORγt	+	+	-	-	Still have NALT (Eberl et al., 2004)
RORα	+	-	+	+	Normal (Wong et al., 2012)
Nfil-3 (*E4BP4*)	-	+	+	+	Normal (Gascoyne et al., 2009)
Gata-3	Lack thymic NK cells	-	+	+	Normal (Hoyler et al., 2012; Mjosberg et al., 2012)
AhR	+	?	CD4^-^ LTi cells reduced	Reduced	No ILFs and CP Normal PP and LN (Lee et al., 2011)
Tox	Fail to mature	?	Reduced	Reduced	No LNs; reduced size and number PPs >90% reduced ILCs (Aliahmad et al., 2010)
Runx/Cbfβ2	+	?	Reduced	?	Impaired organization Reduced (Tachibana et al., 2011)
T-bet	Immature	+	+	–	Normal

## EARLY DEVELOPMENT OF INNATE LYMPHOCYTES

The progenitors of innate lymphocytes develop first from common lymphoid progenitors (CLPs) in the fetal liver (Sawa et al., 2010; Vonarbourg et al., 2010) which give rise to multiple lymphoid lineages including B cells, T cells, and ILCs. In the fetal liver α4β7^+^ CLPs are able to differentiate into NK cells and all subsets of Rorγt^+^ ILCs. The development of ILCs from these precursors is dependent on the expression of Notch, which is regulated differently in fetal and adult precursors (Possot et al., 2011). α4β7^+^ CLPs are severely reduced in *Id2*-deficient mice indicating that Id2 lies upstream of Rorγt in the developmental pathway (Cherrier et al., 2012). These cells also express the chemokine receptor CXCR6 but this marker is not exclusive to LTi cells (Possot et al., 2011). It is proposed that both CD4^+^ and CD4^-^ LTi cells arise from a Rorγt^+^α4^+^β7^+^ progenitor while NKp46^+^ ILCs emerge predominantly from a Rorγt^+^α4^+^β7^-^ cell fraction (~50% NKp46^+^ ILCs) suggesting they are distinct lineages (Narni-Mancinelli et al., 2011). Challenging this concept, it has also been reported that Rorγt^+^ NKp46^+^ ILCs can arise from LTi cells following adoptive transfer (Vonarbourg et al., 2010). Recently, our group has discovered that NKp46^+^ ILCs arise solely from the CD4^-^Rorγt^+^ ILCs and not the CD4^+^ LTi subset (discussed in more detail below; Rankin et al., 2013). Thus, we provide evidence that CD4^-^ LTi cells and NKp46^+^ ILCs are of the same lineage distinct from CD4^+^ LTi (Rankin et al., 2013). This Rorγt^+^α4β7^-^ subset which gave rise to ~50% NKp46^+^ cells was unable to generate CD4^+^ LTi cells but could make CD4^-^ Rorγt^+^NKp46^-^ (Sawa et al., 2010). Therefore, it may be that this progenitor is the true CD4^-^LTi/NKp46^+^ ILC precursor that colonizes that gut while Rorγt^+^α4β7^+^ cells generate classical LTi cells essential for lymphoid tissue generation.

Natural killer cells, in contrast to LTi cells and NKp46^+^ ILCs do not require Rorγt or IL-7 for their development. Until recently, the earliest committed precursor of NK cells, the NKP, was identified through the lack of expression of pan-NK cell markers such as NK1.1 and CD49b and the expression of IL-2Rβ (Rosmaraki et al., 2001). Our recent development of an Id2-GFP (green fluorescent protein) reporter mouse where GFP is expressed under the control of the endogenous Id2 promoter identified that this population was heterogeneous and only a small fraction of cells expressed Id2 (Carotta et al., 2011; Jackson et al., 2011). This population (lin^-^CD122^+^NK1.1^-^CD49b^-^Id2-GFP^+^) gives rise exclusively to NK cells and has been defined as the pre-pro NK cell. This population has also more recently been identified using the surface marker CD244 and CD27 (Fathman et al., 2011). The induction of Id2-GFP in the pre-pro NK cell identifies the first commitment of progenitors to an NK but not T or B cell lineage. It will be important to determine the identity of the common innate lymphoid progenitor (CILP) and position it in the developmental tree to gain a greater understanding of the Id2-dependent precursors of ILCs.

### INHIBITOR OF DNA BINDING 2

Inhibitor of DNA binding 2 is a helix-loop-helix (HLH) transcriptional regulator that plays diverse roles in directing lymphocyte development and function. It is part of a group of closely related proteins, Id1–4, that all share the highly conserved HLH motif (Sun et al., 1991; Voronova and Lee, 1994). They regulate transcription by inhibiting the function of E-box proteins. E-box (or E) proteins are another class of transcription factor and include the family members HEB, E2-2, and the E2A gene products E12 and E47 (Murre et al., 1989a,b). E proteins are defined by their two highly conserved domains: (i) a HLH domain which regulates homo- or heterodimerization and (ii) a basic domain is important for binding to E-box sequences on the DNA of target genes (CANNTG; Murre et al., 1989b). Id proteins are able to inhibit E protein function by forming a heterodimer with their complementary E protein *via* their common HLH domains. As Id proteins lack the basic domain of E proteins, the Id/E protein heterodimer is unable to bind to DNA and thus transcription is blocked (Sun et al., 1991; Voronova and Lee, 1994). In addition to binding to E proteins, Id proteins have also been shown to interact with other transcription factors including retinoblastoma protein (Rb), the ETS (E-twenty six) and Pax (Paired Box) families (Iavarone et al., 1994; Yates et al., 1999; Roberts et al., 2001).

Inhibitor of DNA binding 2 regulates a diverse number of cell fate decisions during lymphopoiesis. It is involved in the development of effector CD8^+^ T cells, DCs, NK cells, and ILCs (Yokota et al., 1999; Hacker et al., 2003; Cannarile et al., 2006; Jackson et al., 2011; Rankin and Belz, 2011). Genetic ablation of Id2 in mice results in the complete failure to develop lymph nodes (LNs), Peyer’s patches, and other secondary and tertiary lymphoid tissues including the NALT which is not affected by loss of other transcription factors such as Rorγt (Yokota et al., 1999). Loss of the ability to form these lymphoid tissues has been attributed to the lack of LTi cells in these mice although multiple ILC populations are absent and could impact on development (Yokota et al., 1999; Cherrier et al., 2012). *Id2*^-/-^ mice also express a marked reduction in immature NK (iNK) cells in bone marrow and spleen (Boos et al., 2007). In this setting, Id2 acts to inhibit the transcriptional program induced by E proteins and directs progenitors toward an innate cell lineage as deletion of E2A was able to partially overcome the need for Id2 in LTi cell development and restore the development of lymphoid tissue. In addition, NK cell development in the bone marrow was also restored, however, splenic NK cells were poorly rescued, with only a small number of residual cells which exhibited a thymic rather than splenic NK cell phenotype (Boos et al., 2007). Whether expression of Id2-GFP in an early progenitor also marks the proposed CILP similar to the pre-pro NK cell remains unclear.

### Runx/Cbfβ COMPLEX

The Runx/Cbfβ complex has been shown to be involved in the early differentiation pathway of LTi cells (Tachibana et al., 2011). The Runx complex consists of a DNA binding subunit, Runx1, 2, or 3 and a non-DNA binding partner Cbfβ that increases the affinity of the Runx subunits for DNA. Runx is essential for the differentiation of several hematopoietic cell lineages including B cells, NK T cells, and CD8^+^ T cells (Okuda et al., 1996; Ichikawa et al., 2004; Egawa et al., 2005; Setoguchi et al., 2008). Mice lacking either *Runx1* or *Cbfβ* have defects in lymphoid tissue organogenesis (Tachibana et al., 2011). Runx1/Cbfβ complexes regulate LTi cell differentiation at distinct stages prior to and following Rorγt expression. In both *Runx* and *Cbf*-deficient fetuses, there was a significant reduction in the frequency of lin^-^α4β7^+^IL-7R^high^Rorγt^+^ LTi precursor cells, a phenotype that parallels that observed in Id2 and Rorγt-deficient mice (Tachibana et al., 2011). In contrast to Id2 and Rorγt loss, an even earlier, more pluripotent precursor, the lin^-^α4β7^+^IL-7R^int^Rorγt^-^ population is reduced in the absence of *Cbfβ*. Thus, Runx1/Cbfβ2 complexes impair LTi cell development prior to the induction of the Id2 and Rorγt transcriptional programs. Given this finding, it might be expected that NK cell development would also be significantly affected by loss of Runx1/Cbfβ complexes. However, NK cells appeared normal in the absence of Runx1/Cbfβ, most likely because NK cells and CD8^+^ T cells dominantly express Runx3, rather than Runx1 (Ohno et al., 2008). In contrast, Cbfβ is required for NK cell development (Guo et al., 2008). In the absence of Cbfβ, lin^-^ fetal liver cells produce very few CD122 (IL2Rβ)^+^ cells capable of responding to IL-15 which is necessary to generate NK cells. CD122, a subunit of the IL-15R, is directly regulated by Cbfβ (Ohno et al., 2008). Nevertheless, transplantation of *Cbfβ*-deficient bone marrow into irradiated recipient adult C57BL/6 mice revealed that NKP cells could be detected in spleen indicating that NK deficiency occurred at the NKP to iNK cell transition, whereas in fetal liver, NKP could not be detected and thus the block in NK development in the fetus must occur prior to the emergence of NKP.

## NATURAL KILLER CELLS (ILC1)

Natural killer cells form part of the immediate response to pathogens or antigens expressed by transformed (i.e., cancerous or stressed) cells and thus are critical during the innate and adaptive immune responses (Vivier et al., 2011). They are distinct from adaptive cells as they do not express somatically re-arranged antigen-specific surface receptors but instead respond through a repertoire of activating and inhibitory receptors (for example, Ly49 molecules in mice, and killer cell immunoglobulin-like receptors (KIRs) in humans; Raulet et al., 2001; Lanier, 2005). The balance between stress-induced activation of NK cell receptors (such as the natural cytotoxicity triggering receptor 1, *NCR-1*, encoding NKp46) and major histocompatibility complex class I (MHC class I)-mediated inhibition controls the cytotoxicity of NK cells during pathogen infection and in destruction of tumors.

Natural cytotoxicity receptors are immunoglobulin-like transmembrane glycoproteins which signal through adaptor proteins with intracellular tyrosine-based activation motifs (ITAMs). They are mostly expressed on NK cells. Humans can express NKp30, NKp44, and NKp46 while mice express NKp46 exclusively (Spits and Cupedo, 2012). Gazit et al. (2006) adopted an elegant approach to investigate the role of NKp46 in NK cells by inserting GFP into the *Ncr1* locus. This enabled direct visualization of NKp46 expression on NK cells during influenza infection where NKp46 recognizes the ligand viral hemagglutinin in a sialic acid-dependent manner (Mandelboim et al., 2001). It also showed that NKp46^+^ NK cells that accumulated in the lung were critical for the control of lethal influenza. Using this model it has also been demonstrated that NKp46 expression by NK cells is important in controlling tumors (Gazit et al., 2006) and for the development of type-1 diabetes (Gur et al., 2010). Recently, a loss-of-function mutant of NKp46 (*Noé* mice) has shed light on the mechanism of action of NKp46 (Narni-Mancinelli et al., 2012). *Noé* mice exhibit a point mutation (W32R) that prevents NKp46 expression on the surface, but did not impair intracellular expression. This phenotype was accompanied by increased responsiveness to stimuli and greater resistance to MCMV and influenza infection. Detailed molecular analyses showed that down-regulation of NK cell activity was associated with silencing of the transcription factor *Helios* in the NK cells and attenuation of T cell responses. These studies revealed that NKp46 acts in a dose-specific manner to tune the optimal development of the adaptive immune response.

Murine NK cells require IL-15 signaling through the IL-15R (composed of three subunits, namely IL-15Rα, CD122, and CD132) for their survival (Mrózek et al., 1996; Kennedy et al., 2000; Cooper et al., 2002; Ranson et al., 2003). Although the majority of NK cells are generated in the bone marrow from CLPs, a developmentally distinct subset of NK cells that arise from bi-potent NK/T cell progenitors in the thymus (Vosshenrich et al., 2006). Bone marrow and thymic-derived NK cells can be clearly differentiated as thymic NK cells require the transcription factor *Gata3* for development and are dependent on IL-7, in addition to IL-15, for survival (Vosshenrich et al., 2006). They have an immature phenotype with reduced cytotoxic activity and express lower levels of inhibitory receptors including the Ly49 family and CD94 (Vosshenrich et al., 2006). In the bone marrow, multiple stages of NK cell development have been defined on the basis of their phenotype, function, and proliferative capacity (Hayakawa et al., 2006; Huntington et al., 2007). These subsets can be distinguished by the expression of CD27, CD11b (Mac-1), IL-7R, and KLRG-1, which are differentially regulated through maturation (Brady et al., 2004; Hayakawa et al., 2006). iNK cells express low levels of CD11b and the inhibitory receptors CD94 and the Ly49 family, do not express CD49b (DX5) and exhibit low cytotoxicity (Rosmaraki et al., 2001; Kim et al., 2002). During maturation, NK cells up-regulate CD49b, acquire Ly49 and CD94-NKG2 receptor expression then expand to become Mac-1^high^ (Kim et al., 2002). Once they mature, NK cells migrate to different tissues including the spleen, LNs, liver, lung, and skin where they develop tissue-specific phenotypes and functions. Furthermore, a unique subset of hepatic NK cells has been described, which constitutively expresses Trail and IL-7R but not Eomesodermin (Eomes) develops independently of the bone marrow (Andrews and Smyth, 2010) and persists as a stable lineage in the liver (Takeda et al., 2005). However, the ontogeny and specific functions of these unconventional NK cells is yet to be fully elucidated.

Natural killer cells found in the intestinal environment are present at low frequencies. Functionally and transcriptionally, they are more similar to other conventional NK (cNK) cells than the intestinal ILCs (Reynders et al., 2011). In the intestinal lamina propria, a large proportion of NK cells express the markers IL-7R and c-Kit and do not express Eomes, a phenotype shared by immature cNK cells, thymic NK cells, and liver-derived NK cells. In addition, an unusually high proportion of these NK cells lack the expression of CD27 and maturation markers such as KLRG-1 and CD11b. Further phenotypic analysis of intestinal NK cells has shown they express only low levels of NKG2D, the Ly49 receptors and exhibit low cytotoxic activity, again indicating their immaturity (Sanos et al., 2009). Whether this unique subset of intestinal NK cells are a distinct lineage or alternately are a gut-specific version of thymic or hepatic NK cells has not yet been addressed. Nevertheless, effector molecules such as perforin, granzymes, and IFN-γ produced by NK cells following activation play crucial roles in defense against tumors and viral infections. NK cells are therefore thought to represent the innate version of CD4^+^ Th1 cells and cytotoxic CD8^+^ T cells (Trinchieri, 1989; Sun and Lanier, 2011).

### TRANSCRIPTIONAL REGULATION OF NK CELLS

#### Nuclear factor interleukin-3

Nuclear factor IL-3 is a critical transcriptional regulator for NK cell development, but does not appear to affect the development of any other ILC subsets (Gascoyne et al., 2009; Kamizono et al., 2009). The blockade in NK development occurs at the NKP to iNK transition. Residual NK cells found in *Nfil3*^-/-^ mice have impaired cytotoxic activity and reduced IFN-γ production suggesting that Nfil3 could also be essential for maintaining mature NK cell function (Gascoyne et al., 2009; Kamizono et al., 2009). Nfil3 is induced following IL-15 signaling through the IL-15R and is thought to act prior to the requirement for Id2 in NK cell development (Gascoyne et al., 2009). Indeed, over-expression of Nfil3 results in an increase in the expression of Id2 in hematopoietic progenitors and was sufficient to partially rescue NK cell production from progenitors. Thus, Nfil3 was thought to directly induce Id2 in the NK cells (Gascoyne et al., 2009). This conclusion assumes that Nfil3 and Id2 lie sequentially in the signaling pathway which may not be the case, as loss of Nfil3 resulted in only ~30% reduction in Id2 expression. An alternate possibility is that Id2 and Nfil3 may exert separate critical effects in parallel in these cells.

#### T-box factors T-bet and Eomes

T-bet (encoded by *Tbx21*) and Eomes (encoded by Eomesodermin) are highly homologous T-box transcription factors that play important roles in regulating the function of multiple cell types especially NK cells (Lazarevic and Glimcher, 2011; Gordon et al., 2012). In particular T-bet, like the transcriptional repressor B lymphocyte-induced maturation protein 1 (Blimp1) has been suggested to be involved in maturation and acquisition of effector functions in NK cells (Townsend et al., 2004; Jenne et al., 2009; Kallies et al., 2011). More recently it has been shown that these two transcription factors act in a sequential manner whereby T-bet directs the development of iNK cells and stabilizes the immature phenotype (Gordon et al., 2012). Eomes allows NK expression of a diverse repertoire of Ly49 receptors and maintenance of a mature phenotype (Gordon et al., 2012). In addition, T-bet and Eomes appear to have distinct roles in the emergence of different NK cell subsets. For example, deficiency of T-bet leads to the loss of hepatic NK cells but not cNK cells (Gordon et al., 2012), suggesting a specific developmental pathway for intrahepatic NK cells (Andrews and Smyth, 2010).

#### Ets-1

Ets-1 is a member of the Ets family of winged HLH transcription factors. *Ets-1*^-/-^ mice display significantly reduced splenic NK cells with decreased cytotoxic activity (Barton et al., 1998). The mechanism by which Ets-1 regulates the development and effector function of NK cells has only recently been described. Ets-1 acts at the pre-pro NK stage, directly regulating the expression of Id2 and T-bet (Ramirez et al., 2012). The residual *Ets-1*^-/-^ mature NK cells have reduced expression of activating receptors like NKp46, Ly49D, and Ly49H resulting in lower cytotoxic capacity (Barton et al., 1998). Interestingly, *Ets-1*^-/-^ NK cells also exhibit increased expression of IL-15-regulated genes as well as inhibitory receptors, which is a characteristic of chronically activated NK cells. Thus, Ets-1 controls a broad range of transcription factors and receptors that drive NK cell development and function (Ramirez et al., 2012).

#### Tox

*Tox*, similar to *Id2* and *Ikaros*, plays a role in the development of both LTi and NK cells. Tox was firstly described as essential for T cell development (Wilkinson et al., 2002; Aliahmad and Kaye, 2008) but subsequent studies have shown *Tox*^-/-^ mice to have severe defects in mature NK cells in the bone marrow and peripheral lymphoid tissues (Aliahmad et al., 2010). Furthermore, knockdown or over-expression studies in human hematopoietic stem cells have also shown Tox to be an important regulator of NK cell development in humans (Yun et al., 2011). In the few remaining *Tox*^-/-^ NK cells, Id2 expression was significantly reduced but unlike in *Nfil3*^-/-^ mice, ectopic expression of Id2 in *Tox*^-/-^ bone marrow precursors could not rescue NK cell development (Aliahmad et al., 2010). Thus, loss of Id2 expression cannot fully explain the developmental defect observed in *Tox*^-/-^ mice, suggesting that additional regulatory pathways exist for the development of NK cells.

## NUOCYTES, NATURAL HELPER CELLS, AND INNATE HELPER CELLS (ILC2)

Over the last 3 years, a new family of innate lymphocyte has been identified that are able to initiate Th2-like immune responses (Neill and McKenzie, 2011). These have been referred to as natural helper cells (Moro et al., 2010), innate helper cells (Price et al., 2010) and nuocytes (Neill et al., 2010). These populations are Rorα-dependent ILCs that express CD278 (ICOS), ST2 (IL-33R), and IL-17BR (Scanlon and McKenzie, 2012). Whether these ILCs represent tissue-specific versions of the same cell type is not yet understood, but they can collectively be referred to as ILC2s due to their common production of T helper type 2 cytokines, IL-13, IL-5, IL-4, and IL-6 (Moro et al., 2010; Neill et al., 2010; Price et al., 2010; Saenz et al., 2010). These cytokines can activate mast cells, basophils, and eosinophils, which are important for protection against parasitic infections, and have also been implicated in the development of allergic diseases such as asthma (Neill et al., 2010; Neill and McKenzie, 2011). ILC2s expand and secrete these potent Th2 cytokines in response to the IL-17 family member IL-25 and the IL-1 family member IL-33 (Moro et al., 2010; Neill et al., 2010). Nuocytes and natural helper cells are lin^-^ IL-7R^+^ and express the stem cell markers c-Kit and Sca-1. Natural helper cells were first discovered in clusters of lymphocytes found in adipose tissue termed fat-associated lymphoid tissue (FALT; Moro et al., 2010) while nuocytes have been shown to be essential for the expulsion of helminths during infection (Moro et al., 2010; Neill et al., 2010; Price et al., 2010; Saenz et al., 2010). An IL-33 responsive population of ILC2s located in the lung also secrete IL-13 and IL-5. They regulate airway epithelial integrity and promote tissue remodeling following lung disease such as influenza virus infection (Monticelli et al., 2011). During the development of allergic lung disease IL-9 production further promotes secretion of IL-5 and IL-13 in ILCs (Wilhelm et al., 2011). Interestingly, cytokine production in this setting is dependent on collaboration with the adaptive immune response as IL-2 is required for ILC-derived IL-9 production. In turn, these ILC2 cells promote the generation and expansion of IL-13 producing T cells (Neill et al., 2010). Another significant population of IL-33 responsive ILC2s is also present in the intestine of mice and most likely important for helminth expulsion (Hoyler et al., 2012). A human equivalent of the IL-13 ILC has also been identified in nasal polyps of patients with chronic rhino-sinusitis suggesting a role for ILC2s in the pathology of type 2 immunity-associated diseases (Mjosberg et al., 2011).

### TRANSCRIPTIONAL REGULATION OF ILC2s

#### Gata-binding protein 3

Recent evidence suggests that similarities in the transcriptional programs exist between ILC2 and Th2 cells. Gata3 is required for the differentiation and maintenance of ILC2 and is the fate determining transcription factor for differentiation of Th2 cells (Hoyler et al., 2012). Gata3 belongs to the family of transcription factors that bind DNA at the WGATAR motif (Orkin, 1992). It contains two Gata-type zinc fingers and regulates a number of important aspects of T cell function including the secretion of IL-4, IL-5, and IL-13 (Ho et al., 2009). Mice lacking *Gata3* lack IL-13 producing cells, the majority of which are ILC2 cells that express high levels of Gata3 (Moro et al., 2010; Halim et al., 2012; Liang et al., 2012). These mice phenocopy IL-13-deficient mice and display significantly increased susceptibility to *Nippostrongylus brasiliensis* gut infection. While these observations highlight parallels between Th2 cells and ILC2 cells, NK cells also rely on Gata3 for maturation, expression of homing molecules and production of IFN-γ (Samson et al., 2003). Thus, Gata3 plays an important role in terminal differentiation and acquisition of full effector function of multiple innate lymphocyte populations. However, through a *cis*-acting regulatory element located 280 kb 3′ to the *Gata3* gene that appears to be both T cell and NK cell-specific (Hosoya-Ohmura et al., 2011), therefore, Gata3 was postulated to play an important role in directing the differentiation of individual innate populations perhaps through the expression of cell-specific enhancers. In parallel with these observations it was discovered that human ILC2s also highly express Gata3 and this expression, together with enhancement of signal transducer and activator of transcription 5 (STAT5) activation, was driven by expression of the cytokine thymic stromal lymphopoietin (TSLP; Mjosberg et al., 2012). These findings emphasize the complexity of the emerging transcriptional network guiding ILC development, and in addition, underline the similarities that exist in the regulation of these innate cell types between mouse and man.

#### Rorα

Although ILC2s do not require Rorγt for development, it has recently been shown that the highly related transcription factor Rorα is critical for their terminal differentiation and their capacity to effect immunity against intestinal worms (Wong et al., 2012). This positions Rorα as an essential regulator of the ILC2 lineage downstream from Id2. “Stagger” mice (*Ror*α^sg/sg^) exhibit a spontaneous deletion in *Ror*α that prevents translation of the ligand-binding homology domain of Rorα and provide a novel tool for investigating the biology of Rorα in the immune system (Hamilton et al., 1996). ILC2s are readily expanded *in vivo* by the administration of IL-25. In the stagger mice, injection of IL-25 was unable to expand the nuocyte population in contrast to wild-type littermate controls (Wong et al., 2012). Furthermore, loss of the nuocyte population in the lung mucosal tissues resulted in exacerbated responses to asthma, and in the gut, impaired immunity to helminthes infections such as *N. brasiliensis*.

## Rorγt-DEPENDENT INNATE LYMPHOID CELLS (ILC3)

The Rorγt-dependent ILCs are divided into LTi cells, and a heterogeneous population of Rorγt^+^ ILCs found in the intestinal mucosa. These mucosal Rorγt^+^ ILCs include NKp46^+^ ILCs (NCR^+^ ILC3) and LTi-like cells (NCR^-^ ILC3) populations.

### LTi CELLS AND LYMPHOID TISSUE ORGANOGENESIS

Lymphoid tissue inducer cells were originally reported as an obscure subset of CD4^+^CD3^-^ lymphocytes able to colonize the mesenteric LNs and peripheral LN anlagen in the developing mouse fetus (Kelly and Scollay, 1992; Mebius et al., 1997). It is now known that they are comprised of a heterogeneous population of cells where only a proportion express CD4 and they require signaling through IL-7R for their survival and expansion (Adachi et al., 1998; Yoshida et al., 1999; Finke et al., 2002; Kim et al., 2003, 2008; Lorenz et al., 2003; Mebius, 2003; Eberl et al., 2004; Coles et al., 2006; Satoh-Takayama et al., 2010). They are essential for the formation of lymphoid tissues including LNs and mucosal-associated lymphoid tissues (MALT) which encompasses the NALT, GALT, and BALT.

During fetal development, LTi cells colonize LN and Peyer’s patch anlagen where mucosal addressin cell adhesion molecule-1 (MadCam-1), the ligand for α_4_β_7_, is highly expressed (Mebius et al., 1996). They are the first hematopoietic cells recruited to secondary lymphoid tissue where they use LTα_1_β_2_ to interact with the LTβR expressed on mesenchymal cells at LN and Peyer’s patch anlagen (Yoshida et al., 1999, 2002; Mebius, 2003). Activated mesenchymal cells then express adhesion molecules as well as pro-inflammatory cytokines and chemokines to enable recruitment and organization of lymphocytes in a process similar to inflammation. Tumor necrosis factor activation-induced cytokine (TRANCE) signaling is required for LTi cell accumulation at LN anlagen leading to early embryonic development of peripheral LNs, while the loss of IL-7R leads to an absence in development of both LNs and Peyer’s patches (Adachi et al., 1998; Kim et al., 2000).

### Rorγt^+^ ILCs IN THE INTESTINAL MUCOSA – LTi-LIKE ILCs (NCR^-^ AND NCR^+^ ILC3)

Subsets of Rorγt^+^ ILCs that are developmentally and functionally similar to LTi cells but show distinct phenotypic differences have been described in the intestinal mucosa. These have been termed LTi-like cells. They include NCR^-^ ILC3 which can be further divided into CD4^-^NKp46^-^CCR6^+/-^Rorγt^+^ and CD4^+^NKp46^-^CCR6^+^Rorγt^+^ subsets, and NCR^+^ ILC3s (CD4^-^NKp46^+^Rorγt^+^).

These Rorγt^+^ ILCs are most highly represented in tertiary lymphoid structures of the GALT. CD4^+^ LTi cells are most prominent at birth and appear to be involved in the formation of Peyer’s patches. However, after birth, CD4^+^LTi only make up 15–20% of the NCR^-^ ILC3^+^ populations when the CD4^-^ LTi cells and NKp46^+^ ILCs rapidly expand at the same time as microbial colonization, indicating an important interplay between their development and environmental signals.

### LTi-LIKE ILCs (NCR^-^ ILC3) IN THE GENERATION OF TERTIARY LYMPHOID TISSUE

Secondary lymphoid tissue of the GALT, namely mesenteric LN and Peyer’s patch develop in an organized and programed way involving fetal LTi cells, whereas tertiary lymphoid structures develop in response to the environmental cues from microbiota and surrounding tissue and are thought to involve NCR^-^ ILC3 CD4^-^ or CD4^+^ cells (Lorenz et al., 2003; Eberl et al., 2004; Ota et al., 2011). Here, LTi-like cells are able to induce the development of well-organized lymphoid structures capable of carrying out germinal center reactions and promote immunoglobulin A (IgA) class switching in response to inflammation (Lorenz et al., 2003; Tsuji et al., 2008). They are key effector cells that drive both the size and number of cryptopatches (CPs) and isolated lymphoid follicles (ILFs) in the gut tissues.

### NKp46^+^ ILCs (NCR^+^ ILC3)

A major subset of intestinal Rorγt^+^ ILCs express the surface molecule NKp46 in addition to the transcription factors Rorγt and Id2. NKp46^+^ ILCs rely on IL-7 signaling for survival and are themselves potent producers of IL-22 but unlike NCR^-^ ILC3s do not produce IL-17 (Satoh-Takayama et al., 2008, 2010; Reynders et al., 2011). NKp46^+^ ILCs reside mainly in CPs, but are also found in ILFs, Peyer’s patches and at very low frequencies in the mesenteric LNs (Luci et al., 2009). NKp46^+^ ILCs can be further divided according to their expression level of Rorγt and NK1.1 into Rorγt^hi^NK1.1^lo/-^ and Rorγt^int^NK1.1^int^ populations, however whether these are distinct subsets remains unclear (Luci et al., 2009). The human counterpart for NKp46^+^ ILCs are defined by their expression of NKp44 rather than NKp46 (Crellin et al., 2010). NKp46 was originally presumed to be exclusively expressed by NK cells. It was therefore assumed that NKp46^+^ ILCs were most closely related to NK cells, however, NKp46^+^ ILCs are distinct from NK cells in several fundamental ways. NKp46 is not essential for the development or function of NKp46^+^ ILCs, which contrasts with the important role of NKp46 in NK cells (Satoh-Takayama et al., 2009; Narni-Mancinelli et al., 2011). IL-15 is essential for the differentiation, survival, and activation of NK cells but is not required for the development of NKp46^+^ ILCs, which like LTi cells depend on the cytokine IL-7 (Satoh-Takayama et al., 2009, 2010). Functionally, NKp46^+^ ILCs also differ from NK cells as they produce little IFN-γ, no perforin and show little cytotoxic potential (Satoh-Takayama et al., 2008; Reynders et al., 2011). An exception to this is Rorγt^int^NKp46^+^ ILCs that can be induced to produce IFN-γ during intestinal inflammation and are potent inducers of colitis (Vonarbourg et al., 2010).

#### Rorγt^+^ ILCs during inflammation

In addition to the key role Rorγt^+^ ILCs play in the development of lymphoid tissues in the intestinal mucosa, they are crucial for maintaining epithelial integrity through the production of IL-22 and IL-17, signature cytokines secreted by IL-17 producing Th cells (Wolk et al., 2004; Ivanov et al., 2006; Zheng et al., 2007; Pickert et al., 2009; Takatori et al., 2009; Vivier et al., 2009; Ota et al., 2011). ILCs are the main producers of IL-22 in the intestine following stimulation by IL-23 produced by activated intestinal DCs (Sawa et al., 2011; Cox et al., 2012). Interestingly, at steady state the adaptive immune system represses IL-22 production by ILCs, however this is de-repressed following epithelial damage (Sawa et al., 2011). IL-22 activates the transcriptional regulator STAT3 and triggers production of anti-microbial molecules such as β-defensin, lipocalin-c and Reg family proteins from epithelial cells to limit pathogen survival and dissemination (Liang et al., 2006; Sugimoto, 2008; Zheng et al., 2008; Sonnenberg et al., 2011). IL-17 is a pro-inflammatory cytokine that promotes neutrophil recruitment and activation and is also enhanced by IL-23 (Takatori et al., 2009). In contrast to LTi cells, murine NKp46^+^ ILCs are not known to produce IL-17 (Luci et al., 2009; Sawa et al., 2011) although the complete spectrum of cytokines that this population does produce during inflammation has not been fully assessed. Rorγt is known to regulate IL-17, so it is intriguing that these Rorγt^+^ILCs lack production of this cytokine implying different transcriptional regulation of cytokines in NKp46^+^ ILCs compared with LTi cells. The importance of IL-23 induced IL-22 production by Rorγt^+^ ILCs is demonstrated during experimental models of intestinal inflammation such as *Citrobacter rodentium*, as mice deficient in these cells rapidly succumb to infection (Satoh-Takayama et al., 2008; Zheng et al., 2008). Here, ILC-derived IL-22 can be induced by lymphotoxin-β in the presence of IL-23 and reduces the systemic dissemination of bacteria from the colon to the liver and spleen (Ota et al., 2011; Sonnenberg et al., 2011). While IL-17 and IL-22 are important to maintain intestinal homeostasis and protect against intestinal pathogens, aberrant production of these cytokines has been associated with inflammatory bowel disease and colon cancer. IL-17 produced by ILCs is thought to be one of the key cytokines in driving colon inflammation in an innate model of *Helicobacter hepaticus* driven colitis (Buonocore et al., 2010). Human studies show that IL-17 production and the frequency of ILCs increases in patients with inflammatory bowel disease (Geremia et al., 2011). IL-22 can also promote intestinal disease by enhancing epithelial cell proliferation leading to colon cancer (Huber et al., 2012). Therefore it is important to understand the activating factors and molecular pathways controlling IL-17 and IL-22 production as this will eventually lead to the development of improved treatments for intestinal disease.

NKp46^+^ ILCs are a critical source of IL-22 but despite this, the relative contribution of IL-22 secreted from NKp46^+^ ILCs or other LTi-like cell subsets is not clear. Until recently, the extent to which one population might dominate or compensate for the other may depend on the type of mucosal activation was unclear, however we have recently shown that other Rorγt^+^ cells cannot compensate for the absence of IL-22 producing NKp46^+^ ILCs during *C. rodentium* infection (Rankin et al., 2013).

#### Rorγt^+^ ILCs and commensal microflora

During steady state, Rorγt^+^ ILCs play critical roles in maintaining mucosal homeostasis through responding to carefully regulated signals from the epithelial layer and commensal bacteria in the intestine. Firstly, the induction of ILFs from CPs occurs in response to external stimuli such as peptidoglycan from gram-negative bacteria in the gut lumen (Bouskra et al., 2008). In this situation, B cells and other lymphocytes are recruited to CPs in a lymphotoxin receptor-dependent manner (Lorenz et al., 2003; Bouskra et al., 2008). Interestingly, ILFs do not develop in germ-free mice, but instead remain as CPs (Hamada et al., 2002; Pabst et al., 2006; Bouskra et al., 2008). Therefore, the number of CPs, ILFs and commensal microflora are tightly controlled by a dynamic activatory/inhibitory negative feedback loop (Bouskra et al., 2008). Increased formation of ILFs enhances the capacity of the immune system to target responses toward the luminal bacteria. In turn, this restricts bacterial proliferation and invasion and serves to shut down the signals that induce ILFs (Sawa et al., 2010; Ota et al., 2011; Sonnenberg et al., 2011). Adult LTi cells become activated and induce recruitment of other lymphocytes through a chemokine-mediated network. CCR6 responds to signaling from CCL20 (also known as MIP-3α), a molecule highly expressed by the epithelium overlying Peyer’s patches and ILFs. β-defensin 3 also binds CCR6 and is expressed by inflamed epithelia and intestinal CPs. Thus, it is suggested that Rorγt^+^ ILCs depend on CCR6 responses to CCL20 and β-defensin signaling (Sawa et al., 2010).

Secondly, Rorγt^+^ ILCs constitutively produce IL-22 where the levels are controlled by careful integration of signals from commensal bacteria and the epithelial layer. Vonarbourg et al. (2010) also showed that commensal microflora and IL-7 can act to stabilize Rorγt expression within the NKp46^+^ ILC population, but they apparently do not induce their differentiation. Other reports have shown that intestinal microbiota have no effect on relative or absolute numbers of ILCs in the gut (Reynders et al., 2011; Sawa et al., 2011) while earlier reports showed a reduction in NKp46^+^ ILCs, but not LTi cells in germ-free mice suggesting a role for commensal microflora in directing their development and function (Satoh-Takayama et al., 2008; Sanos et al., 2009; Vonarbourg et al., 2010). It has now been reported that the absence of microflora enhanced the ability of NKp46^+^ ILCs to produce IL-22 (Reynders et al., 2011; Sawa et al., 2011). The microbiota were shown to induce IL-25 expression on epithelial cells which in turn inhibited NKp46^+^ ILC IL-22 secretion (Sawa et al., 2011). As IL-25R is not expressed on Rorγt^+^ ILCs, this must be occurring via an intermediate cell type such as intestinal DCs. While analyses of the contribution of microbiota to the development, function, and proliferation of ILCs has been investigated, virtually nothing is known about how the individual ILC populations might modify the microbiome.

Thirdly, in a recent elegant study, intestinal ILCs were shown to be crucial for the anatomical containment of commensal bacteria during steady state (Sonnenberg et al., 2012). Here ILCs were depleted in Rag^-/-^ mice and a specific species of commensals that reside in Peyer’s patches and mesenteric LNs named *Alcaligenes* disseminated to the liver and spleen. This resulted in systemic inflammation and increased levels of lipopolysaccharide (LPS). As such, ILCs were crucial for the specific anatomic containment of *Alcaligenes* through the production of IL-22.

### TRANSCRIPTIONAL REGULATION OF ILC3

#### Rorγt

The orphan nuclear receptor Rorγt is an important transcription factor involved in lymphocyte development. It belongs to a large family of steroid hormone receptors that include receptors for steroids, retinoids, thyroid hormones, and vitamin D_3_ and are important regulators of development, cell differentiation, and organ physiology (Mangelsdorf et al., 1995). Rorγt is encoded by the *Rorc* gene and encodes two isoforms, Rorγ and Rorγt from distinct promoters (He et al., 1998; Villey et al., 1999). While the mRNA of the first isoform, Rorγ, is detectable in many tissues, the expression of Rorγt is limited to a small number of lymphocyte populations. Rorγt is highly expressed in double positive (DP) thymocytes (Villey et al., 1999; Sun et al., 2000). The thymus of mice deficient in Rorγt is dramatically decreased in size and cellularity (Kurebayashi et al., 2000; Sun et al., 2000). This is mainly due to the reduced number of CD4^+^CD8^+^ DP thymocytes that results in the reduction of mature single positive (CD4^+^CD8^-^ or CD4^-^CD8^+^) thymocytes. Rorγt^-/-^ thymocytes also show increased levels of apoptosis due to the failure of induction of the pro-survival protein Bcl-xL in the absence of Rorγt (Eberl and Littman, 2004). It was therefore proposed that Rorγt prolongs the lifespan for DP thymocytes allowing them a greater chance to undergo positive selection (Kurebayashi et al., 2000). Beyond its role in thymocyte development, Rorγt expression does not appear again in the T cell development program until peripheral CD4^+^ Th cell subsets undergo differentiation. In this population, Rorγt is induced in response to IL-6 and transforming growth factor beta (TGF-β) signaling to drive naïve CD4^+^ T cells toward a Th17 fate characterized by the production of IL-22 and IL-17 (Ivanov et al., 2006). In the absence of Rorγt, naïve CD4^+^ T cells are unable to differentiate into Th17 cells.

Similar to Id2-deficient mice, Rorγt null mice lack secondary lymphoid tissues due to the absence of LTi cells (Sun et al., 2000; Eberl and Littman, 2003). This includes LNs, Peyer’s patches, ILFs, and CPs. However, in contrast to Id2-deficient mice, NALT development remains unperturbed in the absence of Rorγt (Eberl and Littman, 2003). Using the elegant *Rorγt*^GFP/GFP^ mouse strain (which are deficient in Rorγt) it has demonstrated that both LTi cells and all subsets of Rorγt^+^ ILCs in the intestine are dependent on Rorγt for their development while NK cells and nuocytes are unaffected (Eberl et al., 2004; Satoh-Takayama et al., 2008; Luci et al., 2009; Sanos et al., 2009; Moro et al., 2010; Neill et al., 2010; Vonarbourg et al., 2010). Recent microarray and fate mapping experiments on cells expressing the cell surface receptor NKp46 further support the notion that Rorγt is a lineage defining transcription factor for the all ILC3 cells as intestinal NK cells and NCR^+^ ILC3s were shown to represent two distinct lineages both genetically and developmentally (Narni-Mancinelli et al., 2011; Reynders et al., 2011).

#### Tox

In addition to it’s essential role during NK development (above), Tox is also required for the development and maturation of innate lymphocytes (Neill and McKenzie, 2011). *Tox*^-/-^ mice have ~ 90% reduction in LTi cells in the spleen of neonates and NKp46^+^ ILCs are also diminished (Aliahmad et al., 2010). In contrast to mice that lack *Id2*, in the absence of *Tox*, Peyer’s patches still develop but their size and number are severely reduced. Over-expression of Id2 was unable to recover a normal phenotype in the *Tox*^-/-^ LTi cells suggesting that Tox is downstream of Id2. However, such experimental approaches do not excluded that Id2 and Tox may not lie in the same linear pathway, or that Tox may regulate other critical Id2-independent steps in ILC development. In addition, the role of Tox in specific subsets of intestinal ILCs, including NKp46^+^ ILCs, were not investigated in detail in this study. Nevertheless, although Tox and Id2 are both highly influential in regulating the development of NK and LTi cells, Tox appears to act later and differentially affects Peyer’s patches and LN formation (Aliahmad et al., 2010).

#### Aryl hydrocarbon receptor

Aryl hydrocarbon receptor is a ligand-dependent transcription factor that is a member of the basic HLH/Per-Arnt-Sim (bHLH/PAS) family. AhR is a cytosolic transcription factor bound to co-chaperones such as hsp90 and is normally inactive. On ligand binding, it dissociates from its chaperones and translocates to the nucleus dimerizing with the AhR nuclear translocator (ARNT) that results in changes to gene transcription (Burbach et al., 1992). AhR is a sensor of a number of chemicals and environmental toxins including as 2,3,7,8-tetrachlorodibenzo-*p*-dioxin (TCDD) and phytochemicals such as indole-3-carbinol found in cruciferous vegetables such as cauliflower and cabbage. Endogenous ligands such as the tryptophan photoproduct 6-formylindolo-3,2-b-carbazole (FICZ) have also been identified.

Aryl hydrocarbon receptor has been shown to affect the differentiation of regulatory T cells (Tregs) and enhance the production of IL-17 from Th17 cells. Furthermore, AhR is necessary to allow Th17 cells to produce IL-22 (Kimura et al., 2008; Quintana et al., 2008; Veldhoen et al., 2009; Mezrich et al., 2010). Recently mice lacking AhR have been shown to exhibit a significant deficit in CD4^-^ LTi cells and NKp46^+^ ILCs in the intestinal mucosa (Kiss et al., 2011; Qiu et al., 2012). As the Rorγt ILCs were not completely ablated, it appears that AhR is required for the expansion and survival of CD4^-^ LTi cells and NKp46^+^ ILCs following microbial colonization of the intestine after birth rather than their development. CD4^+^ LTi cells were also affected albeit mildly (Kiss et al., 2011). Extending these studies in an elegant set of experiments it was shown that although *AhR*-deficient mice lack ILFs and CPs, they have normal Peyer’s patch development (Lee et al., 2011). Thus, AhR is specifically required in the formation of postnatal lymphoid tissues highlighting the differential requirements of Rorγt^+^ ILC subsets for ILF and Peyer’s patch development. Investigation into AhR target genes required for ILC development showed that inhibition of the Notch signaling pathway, which is regulated by AhR, critically affected ILC populations.

#### Notch

Notch proteins (Notch1–4) are transmembrane receptors that bind to the surface ligands Delta-like or Jagged before induction of proteolytic cleavage to release the Notch intracellular domain (ICN). On release, ICN translocates to the nucleus where it binds to the CSL/RBP-J (CBF-1/RBP-J, Suppressor of Hairless, Lag-1) transcription factor displacing co-repressors and recruiting co-activators of the Mastermind-like family (MAML). Notch signaling in developing lymphocytes is complex (Bray, 2006). In peripheral CD4^+^ T cells, Notch-1 plays an important role in Th1 polarization (Minter et al., 2005). Both Notch-1 and Notch-2 together with the canonical effector RBPJ (recombination signal binding protein for the immunoglobulin kappa J region) act to protect activated cells against apoptosis (Helbig et al., 2012). In innate lymphocytes, Notch-2 was strongly implicated in the development of adult, but not fetal, Rorγt^+^ cells due to the differentiation of progenitors in response to the stromal cell OP9 expressing the Notch ligand DL-4 (Possot et al., 2011). Disruption of Notch signaling, by the deletion of RBPJ, reduced the number of NK46^+^ ILCs in the small intestine though it had only a marginal effect on other Rorγt^+^ ILC subsets and thus ILF and CP development and this was thought to be downstream of Ahr (Lee et al., 2011). The circuitry regulating the differential dependence of Notch in different ILC subsets appears to depend largely on the induction of T-bet (Rankin et al., 2013).

#### T-bet

The role of T-bet in the development of innate lymphocytes has not previously been investigated except in NK cells. Our recent data showed that T-bet was highly expressed by both the Rorγt intermediate and high populations of NKp46^+^ ILCs but not the NKp46^-^Rorγt^+^ ILCs. Indeed, T-bet was found to be essential for the differentiation of NKp46^+^ ILCs (Rankin et al., 2013). Furthermore, T-bet was crucial for the transition from CD4^-^Rorγt^+^ LTi cells into Rorγt^+^NKp46^+^ ILCs and this transition depended on Notch signaling. Interestingly, both ILC subsets were strongly affected by the absence of Ahr, indicating that CD4^-^ LTi cells are a lineage distinct from CD4^+^ LTi cells (Kiss et al., 2011). Similar to NK cells, Blimp1 was also exclusively expressed by NKp46^+^ ILCs but was not required for their development (Rankin et al., 2013). In contrast to NK cells, we did not observe any expression of Eomes in NKp46^+^ ILCs or LTi cells indicating that Eomes and T-bet operate in a non-redundant manner in this lineage. This identifies T-bet as a master regulator of NKp46^+^ ILC differentiation and the CD4^-^ LTi subset as their direct precursor through the T-bet-Notch signaling pathway (Rankin et al., 2013).

## CONCLUSION

In the past 3 years, it has been clear that innate lymphocytes comprise a heterogeneous group of cells composed of individual populations with discrete, yet complementary functions in maintaining tissue homeostasis and providing protection during an immune responses.

Teasing apart the intrinsic and extrinsic regulatory circuits that result in the development and functional contributions of the various innate lymphocyte populations during inflammation or infection is both an exciting and evolving story. Significant progress has been made in identifying some of the key transcriptional regulators required for the differentiation of ILC subsets, but how they are regulated remains unclear. For example, whether Id2 lies up- or downstream of Nfil3 will require re-examination; or where the terminal differentiation of Rorγt-dependent lineages lies, especially where LTi cells branch off from NCR^+^ and NCR^-^ ILC3s. This may also facilitate further understanding of whether different phenotypes of cells within an ILC population represents a unified lineage that differs because of localization in the body, or they are related but distinct populations within a lineage (e.g., understanding the relationship between the highly similar nuocytes and natural helper cells). Future studies will be important in dissecting these pathways and in understanding the specific contributions ILC subsets make to immunity and pathology in different inflammatory settings.

In the adult tissues, particularly the gut, how the different subsets of Rorγt^+^ ILCs are developmentally related has remained contentious. It is possible that NKp46^+^ ILCs develop as direct progeny of LTi cells or alternately they may arise from a closely related but distinct cell type. Our recent data show that NKp46^+^ ILCs are in fact the direct progeny of CD4^-^ LTi cells isolated from the small intestinal lamina propria. However, in a separate study, LTi cells cultured *in vitro* under various conditions were unable to be induced to express NKp46 supporting that the adult phenotypes of gut ILCs remain stable (Sawa et al., 2011). This may be because the conditions used in these experiments were not optimal to induce differentiation. Nevertheless, a highly specific set of signals from the environment and neighboring cells that induce transcription factors such as T-bet, Notch, Ahr, and other signaling molecules drive the differentiation of NKp46^+^ ILCs. However, in order to understand how innate lymphocytes are regulated in the gut during steady state and disease to effect protective immunity, it will be essential to elucidate further the developmental and micro-environmental requirements of the ILC family. In addition, the degree of plasticity between the various Rorγt^+^ populations is not yet clear. It will be important to uncover whether NKp46^+^ ILCs are a terminally differentiated cell type or, as shown for Th cell subsets, they are in dynamic equilibrium with CD4^-^ LTi cells responding to environmental cues.

A third key question for the field revolves around determining the identity of the CILP as well as the molecules and steps involved in lineage branching to form the different ILC subsets. Id2 is an essential core regulator of the development of the innate lymphocyte family and although the exact identity of the CILP remains to be elucidated it will most likely be an Id2 expressing cell.

Finally, although it is widely recognized that Id2 and Rorγt are essential regulators of a number of ILC populations, the downstream molecular targets of these transcription factors in the LNs and Peyer’s patch have not yet been identified. At a cellular level, elucidating whether the attributes of different ILC populations are shared or distinct, whether there exists plasticity between the populations and whether they develop locally from precursors, or alternately are recruited through the blood will be important in understanding how ILCs orchestrate robust mucosal protection and provide insight into potential avenues to harness and manipulate these cells to promote or ameliorate immune responses.

## Conflict of Interest Statement

The authors declare that the research was conducted in the absence of any commercial or financial relationships that could be construed as a potential conflict of interest.

## Abbreviations

CP: cryptopatch
IL: interleukin
ILC: innate lymphoid cell
ILF: isolated lymphoid follicle
LN: lymph node
NCR: natural cytotoxicity receptor
NK cell: natural killer cell
Ror: retinoic-related orphan.
